# UPLC-Tandem Mass Spectrometry for Quantification of Busulfan in Human Plasma: Application to Therapeutic Drug Monitoring

**DOI:** 10.1038/s41598-020-65919-9

**Published:** 2020-06-02

**Authors:** Kamal M. Matar, Salem H. Alshemmari, Samar Refaat, Alia Anwar

**Affiliations:** 1 Department of Pharmacology & Therapeutics, Faculty of Pharmacy, Kuwait University, Kuwait, Kuwait; 2 Department of Medicine, Faculty of Medicine, Kuwait University, Kuwait, Kuwait; 3 Department of Medical Oncology, Kuwait Cancer Control Center, Kuwait, Kuwait; 4 Department of Pharmacology & Toxicology, Faculty of Medicine, Kuwait University, Kuwait, Kuwait

**Keywords:** Biochemistry, Biochemistry, Oncology, Oncology

## Abstract

Busulfan (Bu) is an alkylating agent commonly used in preparative regimens for hematologic malignant and non-malignant patients undergoing hematopoietic stem cell transplantation (HSCT). The objective of the present study was to develop an UPLC-MS/MS method for quantification of Bu in human plasma. A total of 55 patients with hematologic malignancies (n = 34) and non- malignancies (n = 21) received myeloablative Bu therapy prior to HSCT. A tandem mass spectrometric method was developed and validated to quantify Bu levels in these patients. The method was fully validated over the concentration range of 25–2000 ng/mL (*r* > 0.99). The assay method demonstrated good precision and accuracy. Stability studies indicated that the drug was stable in various conditions. Incurred sample reanalysis findings were within acceptable ranges (<15% of the nominal concentration). Based on the 1^st^ dose AUC results, one third of hematologic malignant patients and half of non-malignant patients needed dose adjustment. However, in subsequent doses (5^th^, 9^th^, and 13^th^), 77%, 82% and 82%, respectively, of hematologic malignant patients and 71%, 67% and 86%, respectively, of non-malignant patients achieved the target range of Bu AUC. The suitability of the developed method for routine TDM of Bu in HSCT patients was demonstrated. The study suggests that the pharmacokinetic profile of Bu varies in both groups.

## Introduction

Busulfan (Bu) is an alkylating agent commonly used in patients being prepared for hematopoietic stem cell transplantation (HSCT) for various types of hematologic malignancies such as acute myeloid leukemia (AML) and myelodysplastic syndrome (MDS) in addition to non-malignancies (thalassemia)^1,2^. In the clinical doses, Bu is considered as a potent cytotoxic drug which causes severe and prolonged myelosuppression.

Bu demonstrates substantial inter- and intra-individual variability in its pharmacokinetic parameters principally the area under the plasma concentration-time curve (AUC)^3^. The intravenous Bu is widely used in the conditioning regimen for HSCT^4^ and its therapeutic efficacy and toxicity are associated with the AUC or the mean blood concentration at steady-state^5^. Generally, an AUC_0-∞_ of 900–1500 μM.min after a single i.v. infusion of 0.8 mg/kg in a 16-dose regimen is correlated with satisfactory HSCT outcomes^6^. A high AUC (>1500 μM.min) is related to a serious risk of hepatic sinusoidal obstruction syndrome (SOS), particularly in the situation of allogeneic transplantation^7,8^. However, at suboptimal AUC (<900 μM.min) values the patient is at increased risk of graft rejection^9^. By virtue of substantial intra-and inter-individual variabilities accompanying Bu pharmacokinetic profiles in addition to its narrow therapeutic index, therapeutic drug monitoring (TDM) for Bu is warranted.

In adults, the protein binding of Bu to plasma albumin ranges between 2.7–55%^10^ and its Vd is in the range of 0.5–0.6 L/kg^11–13^. Moreover, Bu is highly metabolized in the liver by conjugation with glutathione (catalyzed by glutathione-S-transferase enzymes) to form inactive metabolites and then metabolized by cytochrome P450 enzymes^14,15^. About 2% of Bu dose is excreted as unchanged drug in the urine and the main urinary metabolite is methane sulfonic acid^10,11^. Bu elimination half-life was reported in the range of 2.3–3.4 h^16,17^.

Some of the chromatographic assays have been reported for quantification of Bu in biological fluids. The methods comprise high performance liquid chromatographic assays (HPLC)^18–20^, liquid chromatographic-mass spectrometric (LC-MS)^21,22^ and tandem mass spectrometric (LC-MS/MS) assay methods^23–32^. There are some limitations pertaining to the reported HPLC assay methods which include complications such as tedious, arduous and time-consuming sample extraction procedures. In addition, some of the reported HPLC methods derivatize Bu to enhance its detection because of its weak UV absorptivity^18–20^. Derivatization generally leads to prolonged sample pre-treatment procedure which is inappropriate for high-throughput method of analysis as well as it is expensive. On the other hand, the drawbacks of the reported LC-MS methods encompass lack of specificity since some of the reported assays employed selected (single) ion monitoring (SIM) instead of multiple reaction monitoring (MRM) modes^21,22^. The advantage of using triple quadrupole (MSMS), as in the present method, over single quadrupole (MS) systems is that the former methods provide higher selectivity (due to double mass filtering) than the latter, resulting in less interference from co-eluting compounds and matrix components. In addition, more trustworthiness in detection of analytes using MRM in contrast to SIM methods. Therefore, methods using MRM are highly specific, selective and sensitive in quantifying of analytes with complex matrices such as plasma. Other reported methods utilized drugs such as glipizide (anti-diabetic medication) as an internal standard, which may potentially lead to under-estimation of Bu levels in patient’s sample containing Bu and glipizide^24^. The other disadvantages of the reported methods include lack of reliability since they did not assess the potential matrix effect (ME)^21–23,29–32^. Evaluation of ME in LC-MS or LC-MS/MS bioanalytical methods is essential since it may affect the precision and accuracy of the bioanalytical methods and therefore, any data elicited from a method where ME was not assessed may not be thrust-worthy.

The objective of the present study was to develop and validate a rapid, reliable, accurate and reproducible UPLC-tandem mass spectrometric method for quantification of Bu in human plasma. The proposed assay method will be routinely utilized in TDM of Bu by analysis of plasma samples of patients on Bu dosing regimen. Other objectives include comparative assessment of Bu pharmacokinetic profiles in hematologic malignant and non-malignant patients being treated with Bu therapy.

## Materials and methods

### Chemicals and reagents

Busulfan was purchased from Sigma-Aldrich Company (St. Louis, MO, USA) and the internal standard (IS), Busulfan-d_8_, from AlsaChim (Strasbourg, France). Water was purified using a Milli-Q water device (Millipore, Bedford, MA, USA). Human plasma was kindly donated by the Central Blood Bank, Ministry of Health, Kuwait. All other chemicals and reagents were of analytical grade and solvents were of HPLC or LC-MS grades (all were purchased from Sigma-Aldrich Company).

### Instrumentation

Acquity UPLC system (Waters Assoc., Milford, MA, USA) coupled to a triple quadruple mass detector (TQD) was used for analysis of Bu plasma samples. The analytes were separated with Acquity UPLC BEH C_18_ column (2.1 × 50 mm, 1.7 μm) at ambient temperature. The analytical column was equipped with VanGuard pre-column filter of the same packing material (Waters Assoc., Milford, MA, USA). The mobile phase consisted of methanol: 20 mM ammonium acetate buffer (90:10, *v/v*) and delivered at a flow rate of 0.3 mL/min to a positive electrospray ionization interface (ESI^+^) of TQD (Waters Assoc., Milford, MA, USA). Tuning parameters of MS and MS/MS were adjusted by directly infusing solutions of Bu and IS (prepared in the mobile phase) into the ionization probe at a flow rate of 10 μL/min using Hamilton syringe. The ion source and desolvation temperatures were fixed at 150 °C and 350 °C, respectively. The capillary voltage was set at 3.08 kV, cone voltage at 15 V, collision energy at 12 eV and collision cell pressure at 1.000831e^−4^ mbar. The MRM transitions for quantification of Bu and IS were maintained at *m/z* 263.7 > 151.1 and *m/z* 272.2 > 159.1, respectively. Data acquisition, handling and overall instrument control were accomplished employing MassLynx Software (Version 4.1, Micromass, Manchester, UK).

### Standard solutions, calibration standards and quality control samples

Stock solutions of 1.0 mg/mL of either Bu and the internal standard (busulfan-d_8_) were prepared by dissolving an accurate amount of each powder in acetonitrile. Aliquots of both Bu and the IS stock solutions were diluted with 50% acetonitrile/water to provide the corresponding working standard solutions of 50 μg/mL and 5 µg/mL, respectively. The calibration standards were prepared by spiking drug-free (blank) human plasma with Bu to provide concentrations of 25, 250, 750, 1000, 1500 and 2000 ng/mL. Similarly, quality control (*QC*) samples were prepared in blank human plasma at concentrations of 50, 500, 1250 and 1750 ng/mL. The spiked plasma samples were aliquoted (250 µL) into Eppendorf polypropylene tubes and stored at −80 °C pending analysis.

### UPLC-MS/MS assay procedure

Before the assay, frozen human plasma samples involving calibrators, *QC* samples or patient samples were thawed at ambient temperature. A 200 µL aliquot of each plasma sample was transferred to a 15 mL tube and then 25 µL of IS (5 µg/mL) was added and vortex-mixed for 30 sec. To each tube, 300 µL of saturated NaCl solution was added and vortex-mixed for 30 sec. A 4 ml of tert-butyl ethyl ether was added and vortex-mixed for 30 sec. The tube was shaken at 50 rpm for 15 min and then centrifuged at 9000 ×*g* for 10 min at ambient temperature. The organic layer was separated and evaporated under a gentle stream of purified N_2_ gas and then reconstituted with 150 µL of mobile phase and centrifuged at 9000 ×*g* for 5 min. A 10 µL of the clean sample was transferred and injected into the UPLC-MS/MS system.

### Assay validation

The present assay method was validated based on standard international guidelines according to the criteria of industrial guidance for bioanalytical method validation of Food and Drug Administration^33^ as well as others^34^.

### Linearity

The linearity of the present assay method was assessed by spiking Bu in blank human plasma at six non-zero calibrators covering the range of 25 to 2000 ng/mL and then analyzed in replicates of twelve over several days. Bu concentrations were plotted versus the detector responses to obtain the slope, intercept, and correlation coefficient (*r*) of the linear line employing the least squares’ linear regression method using MassLynx software. The precision (determined as relative standard deviation; RSD, %) of the calibration standards should not deviate by values of more than 15% except the lower limit of quantification (LLOQ) which should not exceed 20% of the nominal concentration^33^.

### Accuracy (bias) and precision

Quality control (*QC*) samples of Bu were prepared in blank plasma at concentrations of 50, 500, 1250, and 1750 ng/mL (covering low, medium and high ranges of the calibration standards) and were measured in ten replicates to evaluate the intra-and inter-run precision and accuracy. The intra-run precision was determined from ten replicate analyses of *QC* samples from one calibration curve batch in one day^34^. However, the inter-run precision was determined over a period of four weeks^34^. The RSD, % was considered as a measure of precision whereas, accuracy (or bias, %) was the percent of deviation from the nominal concentration. The precision (RSD, %) determined at each concentration should be ≤15%^33^.

### Selectivity

The selectivity of the present method was assessed by analyzing six independent sources of blank human plasma samples for possible interferences with endogenous components. In this regard, heparinized, hemolyzed and lipemic plasma samples were assessed^33^. On the other hand, some of exogenous compounds at concentrations of 1.0 µg/mL were tested for potential interferences with Bu. The investigated drugs were phenytoin, ethosuximide, oxcarbazepine, lamotrigine, acetaminophen, metoclopramide, domperidone, metronidazole, diclofenac, nicotine, levetiracetam, zonisamide, chloramphenicol, mefenamic acid, and lormetazepam. The mass detector response (peak area) of various plasma samples’ extracts as well as exogenous compounds (at the retention times of Bu and IS) were compared to that of the spiked blank human plasma samples at the LLOQ^33^.

### Stability

The stability of Bu in human plasma sample was evaluated by several studies^33,34^. Freeze-thaw stability was evaluated employing five freeze-thaw cycles from −80 °C to room temperature. At each cycle, QC plasma samples were stored frozen for at least 12 h before they were taken out for thawing at the bench top. The QC samples were kept at room temperature for 2 h to allow for complete thawing before analysis^33,34^. On the other hand, Bu stability in the autosampler was evaluated by storing the processed Bu samples in the autosampler and then the samples were injected at designated time intervals for up to 24 h^33^. In addition, bench-top stability was evaluated by keeping the processed QC samples at room temperature (25 °C) for up to 24 h and the samples were analyzed at various times over that period of time. Moreover, long-term stability assessment was performed by storing the QC samples at −80 °C for 1 month. The samples were analyzed at various times during that period and compared with freshly prepared standard curves and QC samples. The mean value results of stability investigations were calculated and compared to the nominal concentrations^33,34^.

### Recovery and matrix effect

The matrix effect (ME) was evaluated by post-column infusion method^35^. Extraction recovery of Bu from human plasma samples was assessed by using the four *QC* samples. Recovery of Bu and IS was evaluated by comparing the peak areas obtained from blank human plasma samples spiked with the analytes (using the four QC samples) before extraction to those spiked after extraction^33^.

### Incurred sample reanalysis

Incurred sample reanalysis (ISR) was evaluated by re-analysis of some patients’ samples^36^. The original and repeated analyses of patients’ samples were performed employing the present method procedures. The % difference of the original and repeated Bu concentration results was determined as follows^33^:

$$ \% {\rm{Difference}}=100\ast ({\rm{Repeated}}\,\mbox{--}\,{\rm{Original}})/{\rm{Mean}}$$

### Injection carryover

This test was assessed by injecting the upper limit of quantification (ULOQ) of the calibration curve (2,000 ng/mL) followed by blank human plasma sample (0.0 ng/mL). The potential peak areas at the retention times of Bu and IS were compared with the corresponding peak areas of the ULOQ^33^.

### Dilution effect

For Bu plasma samples above the ULOQ, dilution of samples is necessary to provide the concentrations within the calibration curve range and hence the dilution effect is necessary to be assessed. The dilution integrity was evaluated by spiking blank human plasma samples with Bu at concentrations above the ULOQ (e.g., 3, 000 ng/mL) and then the samples were diluted by blank human plasma samples at various ratios involving 1:1, 1:2, and 1:3. Five replicates for each dilution ratio were assessed. The accuracy (bias) and precision (RSD, %) of the diluted plasma samples were determined and compared to the nominal concentrations^33^.

### Clinical application

The present assay method was applied in clinical settings by analysis of Bu plasma samples refereed to our TDM laboratory for routine monitoring of patients taking Bu.

### Patients’ characteristics

A total of 55 patients with hematological (n = 34) and non-hematological (n = 21) malignancies received myeloablative Bu therapy prior to HSCT at Kuwait Cancer Control Center (KCCC). The characteristics and demographics of the patients are presented in Table 1. The present study was approved by Ethical Committees (Ministry of Health and Health Science Center- Kuwait University) as well as approval of KCCC hospital. Informed consent form was not required from the patients because Bu samples were analyzed for routine monitoring prior to HSCT therapy as well as it is a retrospective study (data collected from the patients’ files). None of the research team members had an access to identifying patient information when analyzing the samples or processing the data. All the methods described have been conducted according to Good Clinical Practice and Good Clinical Laboratory Practice guidelines.

**Table 1:** Patients’ characteristics.

Characteristic	Value
**Non-malignant diseases**	
Median age (range)	9 (2–35)
Gender; Males/Females	7/14
Median weight (range)	28.5 (9–74.4)
**Diagnosis, n (%)**	
Thalassemia	15 (71.4)
Aplastic anemia	2 (9.5)
Sickle cell anemia	1 (4.8)
Others	3 (14.3)
**Malignant diseases**	
Median age (range)	35.5 (5–59)
Gender; Males/Females	25/9
Median weight (range)	69.3 (18–105)
**Diagnosis, n (%)**	
AML	13 (38.2)
ALL	12 (35.3)
NHL	3 (8.8)
CML	2 (5.9)
Others	4 (11.8)

Abbreviations: Acute Myeloid Leukemia (AML), Acute Lymphoblastic Leukemia (ALL), Non-Hodgkin Lymphoma (NHL), Chronic Myeloid Leukemia (CML).

## Results and discussion

### UPLC analysis

**Figure 1:**
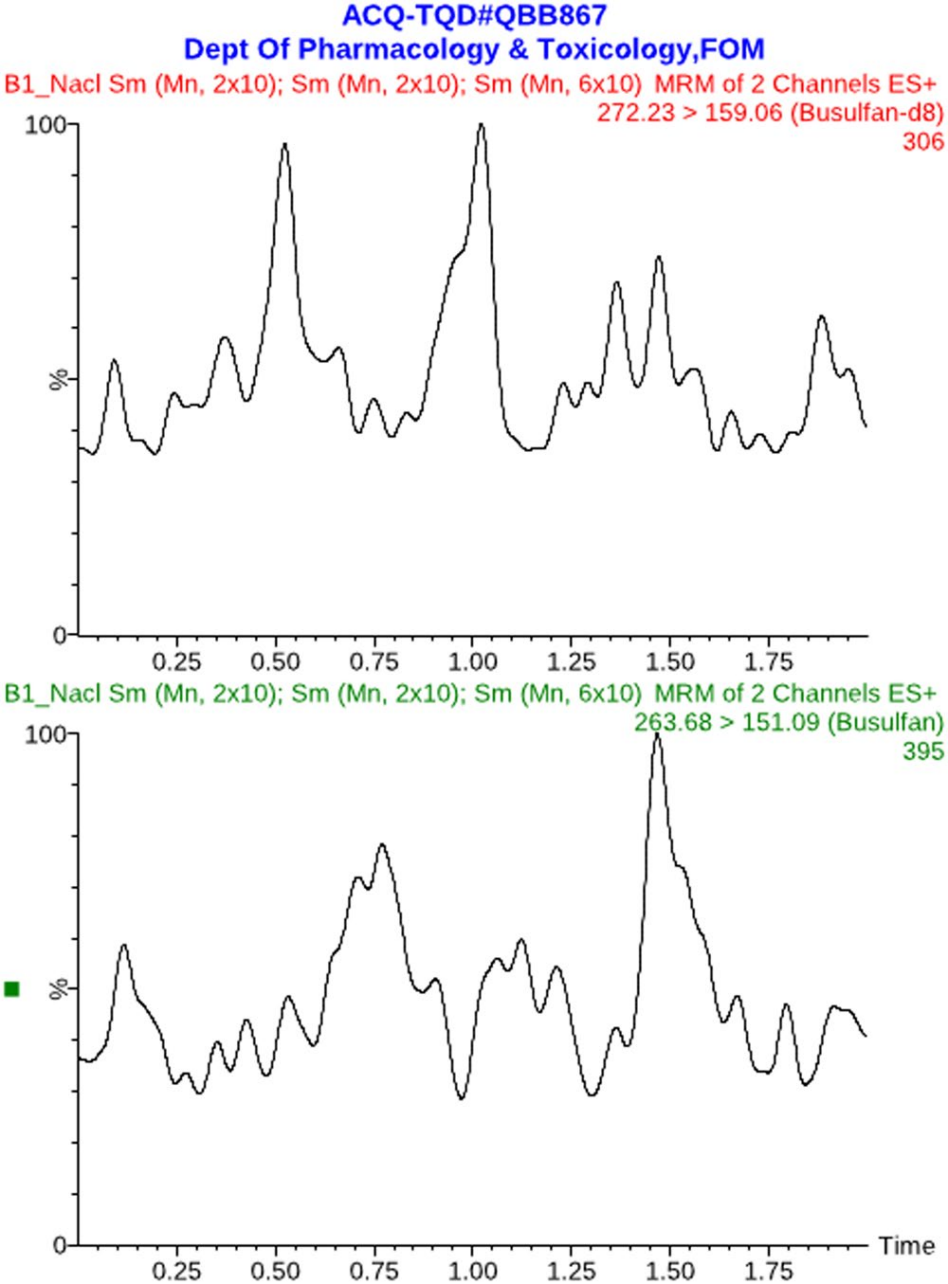
Typical MRM chromatograms of blank human plasma.

**Figure 2:**
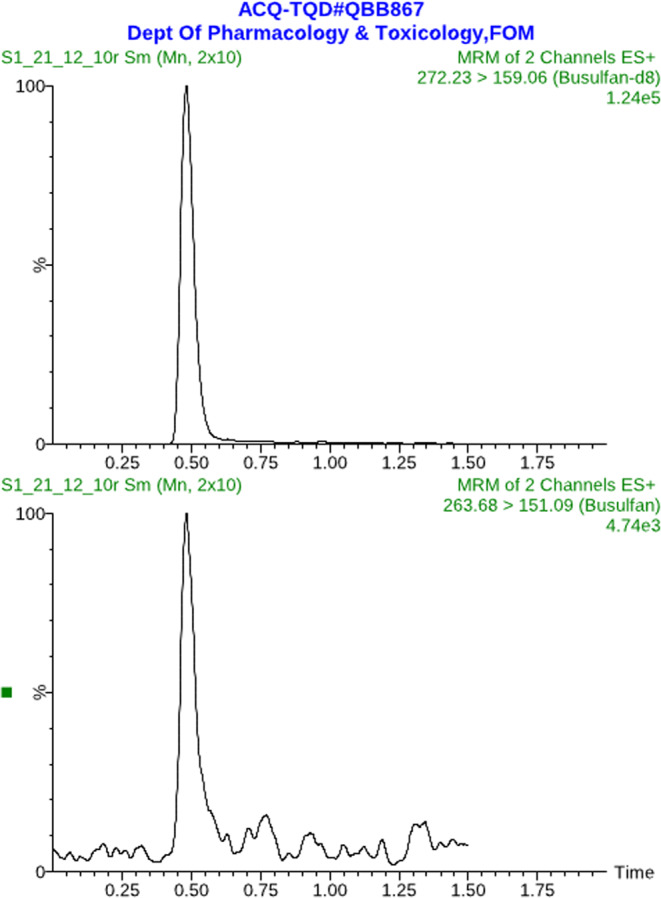
Typical MRM chromatograms of blank human plasma spiked with the analyte (Bu) at the LLOQ level (25 ng/mL).

For obtaining optimized conditions for quantification of Bu in human plasma samples, appropriate tuning parameters were utilized to determine the precursor and product ions of Bu and IS using ESI^+^ mode. The precursor/product ions were determined at *m/z* 263.7 > 151.1 and 272.2 > 159.1 for Bu and IS, respectively. Several endeavors comprising the use of various mobile phase compositions were accomplished to achieve the optimal conditions concerning good resolution and symmetrical peak shapes. The mobile phase consisting of methanol: 20 mM ammonium acetate buffer (90:10, *v/v*) was found optimal for the formation of the precursor and product ions of both Bu and IS. In addition, Acquity UPLC BEH C_18_ column (2.1 × 50 mm, 1.7 μm) equipped with a VanGuard pre-column filter (employing a flow rate of 0.3 ml/min) was found to be the best column for obtaining good peak shape and well separation of both Bu and IS. Using a simple ether extraction step, the total sample pretreatment procedure was very short (approximately 20 min) as well as providing good chromatograms (free of endogenous components) as shown in Fig. 1. The use of an appropriate IS is mandatory for achieving satisfactory method performance. In this regard, deuterated Bu (Bu-d_8_) was utilized for quantification of Bu in human plasma samples. The advantage of this IS is that it has similar physicochemical properties as Bu and hence it compensates for matrix effects since Bu and IS have the same retention times (Fig. 2).

**Figure 3:**
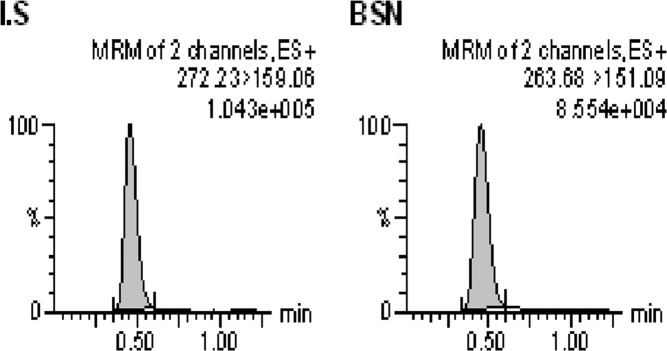
Typical MRM chromatograms of real plasma sample taken 6 h following the 5^th^ Bu dose (19 mg, IV) from a 7 y-old male patient with thalassemia major (Bu Conc = 630 ng/mL).

The selectivity of the present method was assessed by investigating six independent lots of blank human plasma samples involving heparinized, hemolyzed, and lipemic samples. No interferences from endogenous components were observed at the retention times of Bu or IS in blank human plasma samples. In addition, evaluation of interferences from various exogenous compounds demonstrated lack of interferences from the assessed compounds. The current method however, verified good selectivity as shown by lack of interfering peaks from both endogenous and exogenous compounds at the retention times of the drug and IS. Figure 2 depicts typical MRM chromatograms of blank human plasma sample spiked with Bu at the LLOQ (25 ng/mL) and IS. In addition, Fig. 3 demonstrates typical MRM chromatograms of a plasma sample taken from thalassemic child patient who was on Bu therapy.

The present assay method was linear for Bu concentration in the range of 25 to 2000 ng/mL (mean slope = 0.0012, RSD% = 8%, N = 12). Linear correlations (R^2^ > 0.999) were obtained using least squares linear regression method using peak area ratios with the LLOQ of 25 ng/mL (RSD% = 7.4%,). The accuracy and precision of the LLOQ were within the acceptable limits (<15%)^33^.

Intra- and inter-run precision of Bu assay method of four QC levels (50, 500, 1250 and 1750 ng/mL) ranged from 1.23 to 6.52% with accuracy (bias) ranged from −10.66 to −0.24% demonstrating adequate precision and accuracy (Table 2)^33^.

**Table 2:** Intra- and inter-run precision and accuracy for determination of Bu in human plasma by UPLC-MS/MS.

Nominal concentration (ng/mL)	Found (mean ± S.D.) (ng/mL)	RSD, %	^*^Bias, %
^*a*^*Intra-run*			
50	44.67 ± 1.48	3.30	−10.66
500	495.03 ± 6.08	1.23	−0.99
1250	1129.75 ± 21.09	1.87	−9.62
1750	1745.74 ± 21.65	1.24	−0.24
^*b*^*Inter-run*			
50	49.45 ± 3.22	6.52	−1.10
500	489.68 ± 31.20	6.37	−2.06
1250	1213.39 ± 73.16	6.03	−2.93
1750	1714.07 ± 94.06	5.49	−2.05

^*a*^*n* = 10.

^*b*^Precision and accuracy (bias) were determined from 15 different runs over a 4-week period for each concentration.

^*^Bias, % = 100 ×(Found concentration - Nominal concentration/Nominal conc.).

The findings of Bu stability at various situations involving storage of processed samples in the autosampler for up to 24 h and freeze-thaw for five cycles were presented in Table 3. The processed Bu samples were stored in the autosampler (at ambient temperature) and the results demonstrated that the processed samples can remain in the autosampler for up to 24 h without showing appreciable loss in the quantified levels. The results of freeze-thaw stability study demonstrated that the precision ranged between 2.4% to 2.8% and the accuracy ranged between 93.7% to 98.1% (Table 3). The findings exhibited that Bu in human plasma was stable for at least 5 freeze/thaw cycles from −80 °C to room temperature. On the other hand, the findings of Bu stored at −80 °C on its stability demonstrated that the drug was stable at this temperature for at least one month. In addition, storing Bu samples at room temperature indicated that Bu plasma samples were stable for a minimum period of 24 h (Table 3).

**Table 3:** Stability of Bu in human plasma under various storage conditions.

Nominal concentration (ng/mL)	50	500	1250	1750
**Autosampler at 22 **°**C (24 h)**				
Mean conc found (*n* = 8)	54.85	494.68	1274.07	1702.43
RSD, %	8.34	4.38	3.61	2.35
Bias, %	9.7	−1.06	1.93	−2.72
**Freeze-thaw**				
Mean conc. found (*n* = 5)	46.85	484.95	1221.48	1716.5
RSD, %	2.79	2.84	2.37	2.54
Bias, %	−6.3	−3.01	−2.28	−1.91
**Long-term stability at −80 °C (1 month)**				
Mean conc. found (*n* = 6)	51.94	486.63	1231.73	1690.13
RSD, %	10.59	7.58	2.61	3.18
Bias, %	3.9	−2.8	−1.5	−3.4
**Bench-top stability (25 °C for 24 h)**				
Mean conc. found (*n* = 6)	54.85	494.68	1274.07	1702.43
RSD, %	8.34	4.38	3.61	2.35
Bias, %	9.7	−1.1	1.9	−2.7

The ME on the present assay method was assessed by post-column infusion procedure during the method development procedure and consequently the separation system was optimized^35^. The post-column infusion experiment demonstrated that the signals at the retention times of Bu and IS were unchanged when the blank plasma extract was injected into the UPLC-MS/MS system indicating a lack of ion suppression/enhancement using the current assay method.

The findings of the mean Bu recovery from the human plasma samples ranged between 77.9 to 81.7% whereas that of the IS was 82%, indicating the suitability of liquid extraction procedure for separation of Bu from human plasma samples (Table 4).

**Table 4:** Recovery of Bu from human plasma.

Nominal concentration	% Recovery (Mean ± SD)
(ng/mL)	
*Bu*	
50	81.65 ± 3.47
500	77.91 ± 1.05
1250	79.60 ± 3.15
1750	77.91 ± 1.05
*Internal Standard*	81.96 ± 0.54

The findings of incurred sample re-analysis (ISR) by using the present method demonstrated acceptable results which ranged between −13.6% to +13.9% (Table 5). The findings of injection carryover demonstrated that the results were within the acceptable limits (below 15% of the LLOQ of Bu and below 3% for the IS). The dilution integrity of Bu samples above ULOQ assessment demonstrated adequate accuracy and precision (<15% of the nominal concentration).

**Table 5:** Incurred sample reanalysis.

Original Bu conc (ng/mL)	Repeated Bu conc (ng/mL)	Difference	Mean	Difference (%)
4535.8	3957.8	−578	4246.8	−13.61
2017.15	1927.1	−90.05	1972.125	−4.57
1079.3	1194.2	114.9	1136.75	10.11
51.25	47.9	−3.35	49.575	−6.76
3662.5	3789.6	127.1	3726.05	3.41
1859.3	1943.4	84.1	1901.35	4.42
4042.7	4470.8	428.1	4256.75	10.06
1823.2	2095.55	272.35	1959.375	13.90
1057	1101.05	44.05	1079.025	4.08
3863.7	4404.05	540.35	4133.875	13.07
1994.6	2025.85	31.25	2010.225	1.55
1027	1115.35	88.35	1071.175	8.25

The present method demonstrated many advantages over the reported methods such as good precision and accuracy. In addition, the ME was assessed and the results showed lack of ion suppression/enhancement indicating good reliability in Bu detection and quantitation. Moreover, ISR of Bu results were within the acceptable ranges.

### Therapeutic drug monitoring of Bu

Inter-individual variability in the pharmacokinetic profile of Bu has been observed in patients on oral Bu as well as i.v. Bu injection therapy, and the inter-individual variability in metabolic drug handling may contribute to suboptimal outcome^7,37,38^. Monitoring of Bu levels in human plasma samples is necessary for patients on i.v. Bu injection, and dose adjustment based on pharmacokinetic analysis of Bu is commonly experienced. At AUC values of ≤900 μΜ.min, the incidence of graft failure increases, whereas at ≥1550 μM.min there is an increased risk of treatment-related toxicity such as hepatic veno-occlusive disease^7^.

**Figure 4:**
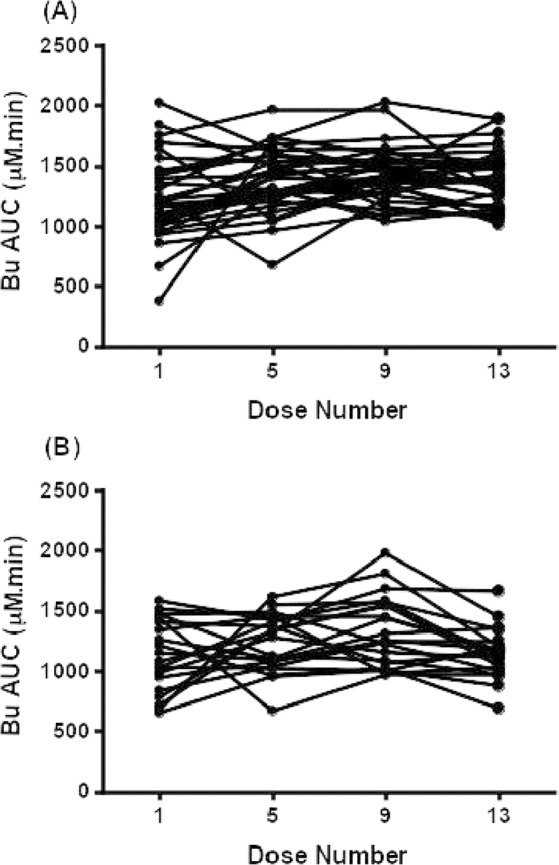
AUC values in (**A**) hematologic malignant and (**B**) non-malignant patients following 1^st^, 5^th^ 9^th^, and 13^th^ Bu dose.

The pharmacokinetic profile of i.v. Bu in hematologic malignant and non-malignant patients using non-compartmental methods are presented in Table 6. Figure 4 depicts individual AUC values following 1^st^, 5^th^, 9^th^, and 13^th^ Bu doses for both groups. Following the 1^st^ Bu dose, the median AUC values in malignant and non-malignant patients were 1181 and 1117 μM.min, respectively, and the median CL values were 2.46 and 3.45 mL/min/kg for malignant and non-malignant patients, respectively. Furthermore, the median Vd values were 0.54 and 0.61 L/kg for malignant and non-malignant patients, respectively.

**Table 6:** Busulfan pharmacokinetics in hematologic malignant and non-malignant patients.

PK Parameter Median (range)	1^st^ dose	5^th^ dose	9^th^ Dose	13^th^ Dose
**AUC (μM.min)**				
Malignant (n = 34)	1181 (381.9–2025)	1361 (680.2–1967)	1405 (1042–2032)	1391 (1021–1895)
Non-malignant (n = 21)	1117 (654.7–1584)	1322 (672.9–1617)	1256 (970.3–1981)	1160 (692.2–1666)
P-Value	0.3868	0.0913	0.181	0.0002
**C**_**max**_ **(ng/mL)**				
Malignant (n = 34)	1073 (634–1710)	1405 (705.2–2135)	1457 (1097–2373)	1405 (1059–2200)
Non-malignant (n = 21)	1158 (822.2–2777)	1389 (692.5–2007)	1404 (1061–2337)	1368 (1020–2118)
P-Value	0.391	0.9111	0.9521	0.8034
**T**_**1/2**_ **(h)**				
Malignant (n = 34)	2.403 (1.687–3.769)	2.71 (1.765–3.998)	2.661 (1.978–4.674)	2.812 (2.012–4.427)
Non-malignant (n = 21)	1.935 (1.417–3.259)	2.243 (1.672–4.165)	2.179 (1.438–3.936)	2.108 (1.547–3.374)
P-Value	0.0017	0.0027	0.0016	0.0001
**Vd (L/kg)**				
Malignant (n = 34)	0.536 (0.375–1.021)	0.4075 (0.236–1.409)	0.385 (0.229–0.917)	0.3705 (0.215–0.931)
Non-malignant (n = 21)	0.606 (0.266–0.949)	0.466 (0.27–1.077)	0.422 (0.245–0.644)	0.4 (0.25–0.632)
P-Value	0.2564	0.0461	0.1315	0.2428
**CL (mL/min/kg)**				
Malignant (n = 34)	2.457 (1.764–4.15)	1.576 (0.903–9.223)	1.563 (0.954–5.356)	1.438 (0.767–5.254)
Non-malignant (n = 21)	3.453 (1.5–6.391)	2.305 (0.93–4.634)	2.444 (0.794–4.051)	2.32 (0.975–4.473)
P-Value	0.0055	0.001	0.0016	0.0009

P-Value < 0.05 is significantly different (Mann Whitney U test).

It has been observed that after the 1^st^ Bu dose to the hematologic malignant patients, 5 patients (14.7%) needed dose increase whereas 6 patients (17.6%) needed dose reduction to achieve an optimal Bu target range of 950–1500 μM.min. However, for the successive dose adjustments, the patients were solely required Bu dose reduction. Following the 5^th^, 9^th^, and 13^th^ doses, Bu dose was reduced in 20.6%, 17.6%, and 17.6% of patients, respectively. On the other hand, following the 5^th^ dose, 2.9% of the patients needed dose increase. However, no increase in Bu dose was needed after the 9^th^ and 13^th^ doses. Similarly, Bu doses were also adjusted for the non-malignant patients. In this regard, after the 1^st^ Bu dose, 5 patients (23.8%) needed dose increase whereas 6 patients (28.6%) needed dose reduction to attain Bu target range of 900–1350 μM.min.

**Figure 5:**
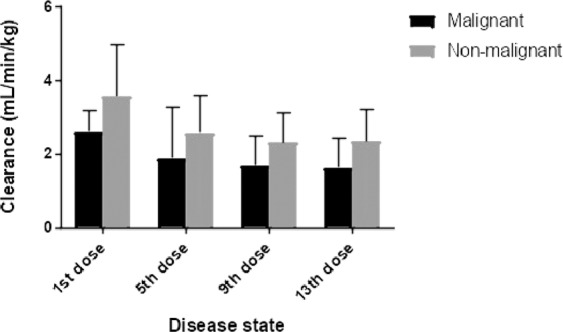
Bu clearance in malignant and non-malignant patients after 1^st^, 5^th^, 9^th^ and 13^th^ dose.

After the initial Bu dose, the median CL in hematologic malignant patients was 2.46 mL/min/kg which was significantly lower (*P* = 0.0055) than that in non-malignant patients (3.45 mL/min/kg). The same trend of significantly lower CL values was seen in both groups in subsequent Bu doses (Table 5 and Fig. 5). Moreover, the 1^st^ dose median CL was significantly high in both groups whereas no significant changes were observed in the CL values in subsequent doses (Fig. 5). This finding concurs with a previous observation in non-malignant children^39^.

**Figure 6:**
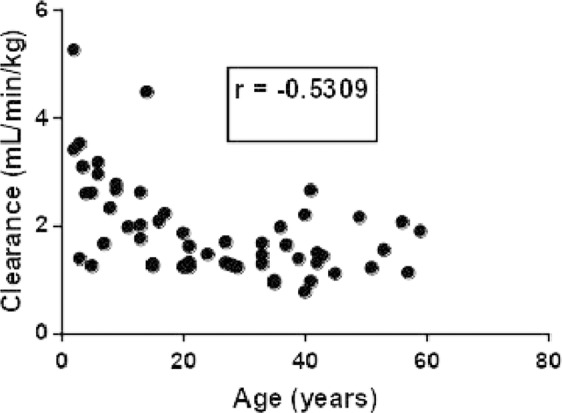
Relationship between Bu clearance and age in hematologic malignant and non-malignant patients (n = 55).

The relationship between age and Bu clearance is depicted in Fig. 6. As shown in the figure, non-malignant patients (mainly pediatrics) demonstrated high Bu CL values in contrast to hematologic malignant patients (mainly adults) indicating that non-malignant patients may require high Bu dose (mg/kg) than hematologic malignant patients to achieve an optimal clinical response. However, this is not the case because the therapeutic range of Bu in non-hematologic patients is lower than that of hematologic malignant patients.

## Conclusions

An accurate, precise, reliable, specific, and reproducible UPLC-MS/MS method for quantification of Bu in human plasma samples is described. The present tandem mass spectrometric method is appropriate for routine analysis of Bu in plasma samples of patients being prepared for HSCT therapy. The described method is routinely employed in our TDM lab for quantification of Bu in human plasma samples of both hematologic malignant and non-malignant patients. Utilization of TDM to Bu therapy minimizes toxicity, maximizes efficacy and improves transplantation outcome. In addition, the pharmacokinetic profile of Bu in hematologic malignant and non-malignant patients have been investigated. To identify the factors that influence the variation in the pharmacokinetic profile of Bu in hematologic malignancies and non-malignancies, population pharmacokinetic studies are warranted.
